# Explainable temporal deep learning for athlete-independent classification of injury-labeled days in competitive distance runners: a methodological benchmark using seven-day training-load histories

**DOI:** 10.3389/fpubh.2026.1936365

**Published:** 2026-08-28

**Authors:** Tianyi Qiao, Wenjun Tian

**Affiliations:** 1 Faculty of Sports, Belarusian National Technical University, Minsk, Belarus; 2 College of Music, Ewha Womans University, Seoul, Republic of Korea

**Keywords:** calibration, distance running, explainable artificial intelligence, grouped cross-validation, injury-labeled athlete-days, integrated gradients, methodological benchmark, temporal convolutional network

## Abstract

**Introduction:**

This study evaluates whether seven-day training-load histories can support athlete-independent classification of injury-labeled days in competitive distance runners. The aim is methodological benchmarking rather than clinical injury-risk deployment.

**Research gap:**

Prior sports-injury prediction studies often use repeated athlete-day observations without fully addressing athlete leakage, rare-event imbalance, probability calibration, and the risk that explainability methods may identify recording artifacts rather than physiological mechanisms. Data and method: The public dataset contained 42,766 athlete-day observations from 74 runners, including 583 injury-labeled observations (1.36%). Seven external-load variables and three internal-response variables were organized from Day −7 to Day −1. A compact dual-stream multi-scale temporal convolutional network with gated fusion and feature-by-day Integrated Gradients was evaluated using athlete-independent five-fold grouped cross-validation. Logistic regression and XGBoost served as baselines, while three ablations and five prespecified neural-network seeds assessed robustness.

**Findings:**

In the primary analysis, DS-MTCN-IG achieved ROC-AUC 0.596, PR-AUC 0.0204, and Brier score 0.01343. Logistic regression achieved a numerically higher ROC-AUC of 0.619, but the paired athlete-cluster bootstrap difference was not statistically significant (ΔROC-AUC = 0.0227, 95% CI − 0.0180 to 0.0714; *p* = 0.291). Across five seeds, the ungated dual-stream model achieved the highest mean ROC-AUC among the deep variants (0.604 vs. 0.598 for the gated model), so the gating hypothesis was not supported. In the non-overlapping evaluation, which removed temporal adjacency between successive prediction targets, DS-MTCN-IG achieved a mean ROC-AUC of 0.469 and did not demonstrate above-chance discrimination. This finding indicates that the weak discrimination observed in the full-window analysis may reflect temporal overlap and autocorrelation between adjacent seven-day histories rather than a stable athlete-independent relationship between training-load history and injury-labeled days. Five-seed Integrated Gradients identified contributions from measured internal-response ratings and availability masks, but exact top-input rankings were unstable across seeds.

**Conclusion:**

The study did not demonstrate a robust or practically useful athlete-independent predictive signal. The non-overlapping null result substantially limits interpretation of the full-window estimates and provides no support for clinical, coaching, or automated injury-warning use. The principal contribution is therefore a methodological audit showing how temporal overlap, participant-aware validation, calibration, model comparison, and attribution stability can alter conclusions in longitudinal sports-injury modeling.

## 1 Introduction

Distance running is an accessible, scalable, and integral part of competitive and recreational running, though with significant burden of running-related musculoskeletal injury. The incidence and prevalence rates are found to be very different across the reviews, as studies differ in populations, exposure denominators, injury definitions and follow-up methods (1). Biomechanical and musculoskeletal factors are also important but prospective evidence is limited and inconsistent for many isolated screening factors, or is injury-specific and not universal (2, 3). These limitations have led to a movement away from the concept of ‘once a week’ screening and toward ‘long term monitoring’ in which risk is seen as a ‘state’ that depends on load, recovery, capacity and previous exposure.

There is not a single workload threshold that can represent running injury. The running-injury framework developed by Bertelsen et al. (4) considers load and capacity as interacting over repeated loading cycles, meanwhile a holistic view of the bone stress injury considers cumulative risk and individual load capacity (5). The concept of network physiology is also consistent with the idea that exercise responses are the result of interacting systems and not singular markers (6). This is immediately applicable to the training of athletes in normal training conditions: kilometers and intensity is external work while exertion and recovery is internal response to the work and perceived training success is the internal response to the external work. Their out-of-orderness might be more informative than either of these streams alone.

With the swift development of wearables and athlete-management technologies, longitudinal monitoring is now technically possible. Validity, interoperability and context of wearables capturing aspects of running gait and training exposure outside the laboratory varies (7, 8). Concurrently, research using machine learning has been used to try to identify patterns of injuries that involve multiple variables that are not captured with the univariable screening. However, as observed in systematic reviews, repeated observations are poorly handled, the studies are small, there is limited external validation, outcomes are heterogeneous and limited, and the studies do not pay sufficient attention to interpretability (9–11). Having a sophisticated algorithm does not, therefore, solve the more basic issues of definition of target, validation design, and clinical usefulness.

The present data are suitable for investigation of these questions as it includes repeated daily observations from competitive runners over a period of about 7 years. The original study demonstrated that machine learning tools could be used to capture a predictive signal from daily and weekly training-load summaries (12), and the public release of the data enables a methodologically reproducible comparison (13). Time-series encoding and deep learning have been used in sports-injury prediction research (14) and multimodal fusion studies have also noted benefits of fusing multiple domains of monitoring (15). The unknown is whether the temporal deep model can be transferred to athletes other than the ones it has been trained against and whether it predominantly learns athlete-specific baselines and patterns of data collection.

This consideration is important because repeated athlete day-rows are not independent. Random row-level splitting may cause observations for the same athlete to be allocated to the training and test sets which facilitates the model to learn from consistent personal traits and temporal trends close to each other. This leakage yields an easier evaluation than the one the designer had in mind when evaluating risk for a new athlete or a truly suspicious athlete. Personalized models are useful if there is enough within-athlete history (16), but serve a different purpose as a generalization from an athlete rather than independently. Clear demarcation of these goals is a key requirement.

A second problem is the rarity of injury-labeled observations. In the present data, only 583 of 42,766 daily records were injury-labeled. Under this prevalence, accuracy is uninformative: a model predicting no injury for every observation would be correct on approximately 98.64% of rows. Precision–recall analysis is more revealing than ROC analysis when the positive class is rare (17), and Matthews correlation offers a balanced summary of the confusion matrix when class sizes differ sharply (18). Calibration is equally important because a probability estimate should correspond to observed event frequency; discrimination alone does not establish usable risk estimation (19).

A third problem concerns explanation. Explainable artificial intelligence is often presented as a route from black-box prediction to actionable prevention. Reviews distinguish post-hoc attribution from causal explanation and warn that an apparently intuitive importance ranking does not establish that changing the feature will change the outcome (20, 21). In sports injury research, this warning is particularly important because predictive variables may encode exposure, athlete identity, recording behavior, or proximity to an outcome rather than a modifiable mechanism. Bullock et al. (22) explicitly cautioned against interpreting predictive features as causal determinants. An explanation method should therefore be used to audit what a model relied upon, not to manufacture clinical causality.

The research problem is consequently methodological as well as substantive. Previous research has demonstrated that training loads can be used for injury identification; however, the literature is inconclusive about the extent to which the training loads carry over to athlete independent validation, whether the external and internal monitoring streams complement each other and whether temporal explanations reveal meaningful load–response relationships or dataset artifacts. The theoretical gap is between reasoning about the dynamic load capacity and its implementation in a testable temporal architecture. The empirical gap is related to the generalization of the results across athletes in a heavily skewed longitudinal data set. The methodological gap is in the integration of grouped validation, calibration, ablation and feature-by-day attribution. The contextual gap relates to transfer to monitoring systems that are not part of the source team, such as Chinese endurance-sport contexts.

China provides a relevant prospective application context, but not the empirical context of this dataset. The National Fitness Program (2021–2025) focuses on mass participation, scientific guidance of fitness and enhanced sport-service systems (23). That agenda could be advanced with data-informed athlete monitoring in universities, provincial teams, running clubs and sports-science institutes. However, a model that has been developed from the Dutch competitive runners cannot be called a Chinese model without local data. Coaching systems, climate, sporting schedules, access to medical staff, language used to rate subjective outcomes, and injury surveillance practices may differ, which will impact both levels of risk and the distribution of predictors. This manuscript thus distinguishes between the development of the model and transfer claims.

The research has three aims. First, it evaluates whether a compact temporal deep-learning model can classify injury-labeled athlete-days from the preceding seven-day history of external-load and internal-response variables under athlete-independent validation. Second, it compares the proposed model with logistic regression, XGBoost, and three neural ablations to determine whether gated dual-stream fusion adds robust predictive value. Third, it uses Integrated Gradients to audit whether the fitted model relies on physiologically interpretable training-load information or on observation-availability patterns created by the recording process.

The corresponding research questions are: RQ1: How accurately can injury-labeled athlete-days be classified for held-out competitive distance runners from the preceding seven-day training-load sequence? RQ2: Does gated dual-stream temporal modeling improve athlete-independent discrimination relative to single-stream, ungated, logistic-regression, and XGBoost alternatives? RQ3: Which external-load, internal-response, and observation-availability inputs contribute most to the fitted model’s scores? Three cautious hypotheses guide the analysis. H1 predicts discrimination above chance and PR-AUC above the observed injury-label prevalence. H2 predicts that dual-stream modeling will outperform single-stream variants, without assuming superiority over conventional baselines. H3 predicts that both measured variables and observation-availability patterns may contribute, while recognizing that attribution is not evidence of injury causation.

The study contributes in four ways. Theoretically, dynamic load–capacity reasoning is used only to motivate the temporal organization of external-load and internal-response histories; the theory itself is not directly tested. Methodologically, it tests all models on disjoint sets of athletes, utilizes metrics suitable for rare events, applies calibration and applies ablation and attribution audits. In practice, it identifies the safeguards required before similar models can be evaluated in prospective athlete-monitoring research in China or other settings. The rest of the article explains the theory, data and analysis, results, implications of these findings, and limits of the findings.

## 2 Literature review and theoretical framework

### 2.1 Running injury as a dynamic load–capacity process

Injury research typically lacks a consistent threshold for determining whether a training session is safe or unsafe. Running-related injuries are highly variable events, and the loading of a tissue is related to biomechanics, intensity, surface, fatigue, recovery and the state of the tissue at the time. The concept of running injury by Bertelsen et al. (4) is that running is a repetitive activity that can cause injury if the load placed on the tissue is greater than its capacity. Willwacher et al. (3) also demonstrated that biomechanical evidence varies based on type of injury, making it impossible to state a “high load equals injury” hypothesis as a blanket rule. This is one reason why the present endpoint is presented as a binary injury, instead of overuse injury: the data is not sufficiently clinical specific to assign observations to a specific tissue pathway. The load–capacity framework is used here only to motivate the temporal organization of predictors. It is not directly tested by the present analysis because the dependent variable is an administrative binary label without diagnosis, anatomical site, onset mechanism, severity, or independent clinical verification. Accordingly, the target is termed an injury-labeled athlete-day throughout the manuscript. Model performance concerns retrospective classification of that source-dataset label, not prediction of tissue injury or empirical confirmation of load–capacity theory.

The load–capacity view also challenges the use of the simple concept of ACWR. Impellizzeri et al. (24) claimed that the theoretical and statistical basis of the ratio is not suitable for causal or prescriptive interpretation. Although training-load metrics may continue to be useful predictors, the association between training load and prediction does not prove a universal training workload rule. Several adaptations have been documented to be produced by increasing the load at low or high intensity during endurance training, which may depend on the athlete’s program or individual (25). The exposure-response trajectory, then, is the relevant object, rather than a single total.

Supporting this interpretation is a dynamic-systems perspective. According to network physiology, adaptation is a process of coordination between the interacting physiological and behavioral systems (6). External training measures record what prescribed/completed, subjective recovery measures record the perceived recovery, and subjective exertion measures record the perceived exertion. Together, these variables are imperfect but they do have a temporal structure which can be a good approximation to changing capacity. The approximation is explicitly empirical: model does not observe tissue load, sleep physiology, nutrition, previous diagnosis, menstrual function, biomechanical loading or psychosocial stress. Both theory and prediction is limited by those unmeasured determinants.

### 2.2 From screening factors to longitudinal prediction

There is little consistency of many biomechanical and musculoskeletal screening variables as noted in prospective reviews (2). This is not to say biomechanics are insignificant; however, group-level associations may not be easily translated to prediction of the individual. Machine learning researchers share the same catch. Van Eetvelde et al. (11) concluded that machine learning can identify nonlinear patterns and potentially high-risk athletes, but methodological quality and model interpretation need improvement. Similarly, Majumdar et al. (26) highlighted the challenges of injury prediction in football due to imbalance, reporting inconsistencies, and longitudinal dependence. New evidence synthesis has validated that external validation and prospective evaluation are still rare (9).

Some methodological precedents are given by several studies, however. Rommers et al. (27) utilized machine learning to evaluate injury risk in elite youth football, demonstrating the role of multivariable pattern recognition but also the impact that the sampled population has on performance. When the athlete’s individual data are available, de Leeuw et al. (16) demonstrated that personalized modeling could contribute to the monitoring of elite volleyball players. Huang et al. (15) fused multiple monitoring domains using a fusion strategy and leveraged interpretability tools to analyze prediction patterns. To promote deep-learning-based sports-injury prediction, Ye et al. (14) transformed training-load time series into images. While supporting models with a temporal and multi-modality perspective, these studies also underscore the need for realistic validation beyond algorithmic novelty. Recent athlete-injury model comparisons continue to illustrate the use of conventional machine-learning baselines, although headline accuracy estimates remain difficult to interpret when outcome prevalence, participant separation, and rare-event metrics are not reported consistently (28). Cross-validation strategy is itself consequential: Kim et al. (29) demonstrated that model performance varied according to record-wise vs. subject-wise validation in an ACL-reinjury modeling context. These studies further support the present emphasis on participant-disjoint validation, transparent baseline comparison, and metrics appropriate for rare outcomes.

The first competitive-runner study, which relied on the same data set and reported moderate discrimination, was conducted by Lövdal et al. (12). This analysis does not aim to replicate the exact preprocessing or split design of the current analysis. Instead, it poses a more stringent generalization question, holding out complete athletes. Consequently, the results of direct AUC comparisons with previously reported data would be misleading. A lower score under athlete-independent validation may reflect a more difficult and more transportable evaluation rather than an inferior implementation.

### 2.3 Explainability, calibration, and decision relevance

Deep models can represent nonlinear temporal interactions, but their flexibility makes auditing essential. Explainable-AI reviews distinguish global feature importance, local attribution, example-based explanation, counterfactual explanation, and mechanistic understanding (20, 21). Integrated Gradients assigns an attribution to each input by integrating gradients along a path from a baseline to the observed input and satisfies sensitivity and implementation-invariance properties (30). For a seven-day sequence, it can produce a feature-by-day map rather than collapsing importance across time.

The relationship of attribution remains dependent on the fitted model, baseline, preprocessing, and correlated inputs. It is not the fact that a feature inflicts harm that leads to a high attribution, but rather that it aids the model in distinguishing records. This is particularly relevant if there is informative missingness or observation availability. An availability mask can be used to encode training participation, if subjective ratings are not provided on rest days. If the recording pattern was attributed a lot, this does not imply that “missing recovery” is a biological risk factor. Use of explanation in this context is diagnostic and hypothesis generating.

Calibration is an added layer of accountability. A model can rank cases with good accuracy and produce too many or too few probabilities. This mismatch can be mitigated by post-hoc calibration techniques like Platt scaling or temperature scaling, but they are estimated without test-data leakage (31). The Brier score is a summary of squared probability error and a calibration curve is a comparison of predicted and observed frequencies (19). As events are rare here, there is significance in any difference in absolute probabilities, and a wide (visually broad) calibration plot with points at 0–1 can mask the region of interest around 0–0.05.

The reporting standards are consistent with these requirements. Data sources, participants, outcomes, predictors, missing data management, model development and validation, and model performance should be described transparently as required by TRIPOD+AI (32). The focus of PROBAST+AI is on risk of bias and applicability of prediction-model studies, such as concerns regarding participants, predictors, outcome and analysis (33). The present design has been arranged in light of these principles: (1) separation of athletes across folds, (2) all test observations kept, (3) reporting of discrimination and calibration, and (4) explicit separation of prediction and causal inference and the documentation of the preprocessing process.

### 2.4 Conceptual framework and hypotheses

There are 2 information streams in the conceptual model. Completed work: session count, total distance, moderate intensity distance, high intensity distance, sprint distance, strength training, alternative training are all represented by the external-load stream. The internal-response stream are perceived exertion, perceived training success and perceived recovery. These streams are processed on a day-by-day basis from Day −7 to Day −1, and the relative importance of the streams is determined by a learned gate. This architecture is motivated by dynamic load–capacity reasoning, but it does not constitute a direct test of that theory because neither tissue capacity nor clinically verified injury is observed. The architectural analysis tests only whether separating and recombining external-load and internal-response histories improves classification of the available injury label.

*H*1: The proposed model will demonstrate discrimination above chance and precision–recall performance above the observed injury prevalence under athlete-independent validation.

*H*2: The gated dual-stream model will outperform external-only, internal-only, and ungated dual-stream ablations because integration permits conditional weighting of exposure and response information.

*H*3: Feature-by-day attribution will identify contributions from both external-load and internal-response streams, although the interpretation will remain predictive rather than causal.

**Figure 1:**
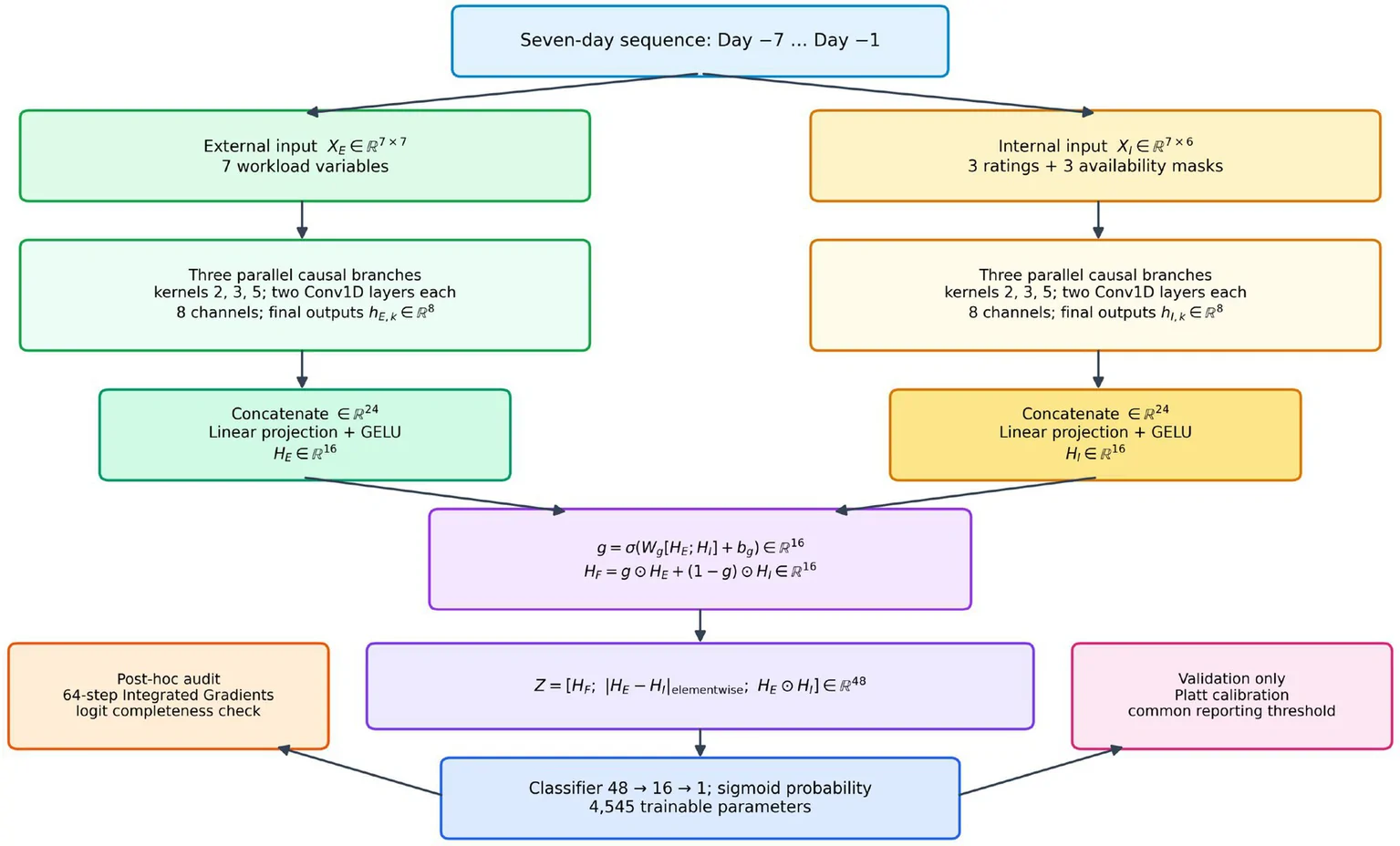
Proposed dual-stream temporal classification and explanation framework. External-load and internal-response sequences are encoded separately from Day −7 to Day −1. The classifier estimates the probability of an injury-labeled athlete-day in the retrospective source dataset. Probability calibration used validation data only, whereas the common descriptive reporting threshold was fixed at the empirical injury-label prevalence. Integrated Gradients were used as a post-hoc audit rather than as causal evidence.

Figure 1 depicts the temporal dual-stream architecture, calibration stage, and explanation procedure. Table 1 summarizes the alignment among the measured endpoint, available predictors, theoretical interpretation, and principal boundaries of inference.

**Table 1:** Topic–dataset–theory alignment and operationalization.

Component	Operational definition	Measurement status	Boundary/interpretive constraint
Dependent variable	Binary injury label	Directly observed	Does not distinguish overuse/acute, site, diagnosis, or severity
External-load predictors	Sessions; total km; moderate/high-intensity km; sprint km; strength; alternative training	Observed over Day −7 to Day −1	Practical workload indicators, not tissue-specific load
Internal-response predictors	Perceived exertion, training success, recovery	Observed proxies	Not validated latent constructs; no SEM/CFA
Mediator/moderator	None	Not estimated	Dataset does not support credible mediation or moderation
Controls	Athlete grouping; fold-specific preprocessing	Design controls	Demographic and clinical controls unavailable
Unit of analysis	Athlete-day sequence	42,766 records	Repeated overlapping windows within 74 athletes
Time period	Approximately 7 years	Source cohort	Numeric date index; calendar date not used as a predictor
Geographic scope	Dutch competitive running team	Source-study scope	China is a prospective implementation context only
Theory	Dynamic load–capacity and multistream monitoring	Motivational framework only	Not directly tested; tissue capacity and clinically verified injury outcomes were not observed.

The study endpoint is an injury-labeled athlete-day supplied by the source dataset. The analysis does not identify injury mechanism, diagnosis, anatomical site, severity, or clinical prognosis. All findings should therefore be interpreted as retrospective classification evidence rather than deployable injury-risk prediction.

## 3 Methodology and data analysis strategy

### 3.1 Research design and data source

The study used a secondary longitudinal prediction design. The source records were released with the article “Injury Prediction in Competitive Runners With Machine Learning” and are available through DataverseNL and a Kaggle mirror (12, 13). The cohort comprised 74 high-level middle- and long-distance runners from a Dutch competitive running team. The records span approximately 7 years and include daily training histories and a binary injury indicator. No Chinese participants are identified in the source data; China is addressed only as a future transfer context.

Two files were audited. The daily file contained 42,766 athlete-day observations and the weekly file contained 42,798 observations. Seventy-four athlete identifiers were present in both. The daily file included 583 injury-labeled rows (1.363%), whereas the weekly file included 575 (1.344%). Across the files, 42,680 athlete-date records overlapped, and no overlapping injury labels disagreed. There were 86 daily-only and 118 weekly-only records. Because the proposed architecture required ordered daily sequences, the daily file served as the primary analytical dataset; the weekly file was used for consistency checking rather than pooled modeling. Thus, the weekly-file consistency check identified no disagreement in injury labels among the 42,680 matched athlete-date records and did not require any alteration of the daily-file modeling dataset.

The unit of analysis was the athlete-day prediction record. Each daily row contained a preconstructed seven-day history with 10 variables per day, yielding 70 longitudinal measurements before availability masks. The source structure labels columns without a suffix as Day −1, suffix 0.1 as Day −2, through suffix 0.6 as Day −7. All model inputs were reordered chronologically from Day −7 to Day −1.

### 3.2 Outcome, predictors, proxies, and unavailable constructs

The dependent variable was the binary injury label supplied in the dataset. Because the file does not contain diagnosis, onset mechanism, anatomical location, clinical verification, or time-loss severity, the outcome cannot be restricted to overuse injury. The manuscript therefore avoids claiming that the model predicts a specific injury mechanism.

The independent variables were grouped theoretically and architecturally. External-load indicators were number of sessions, total kilometers, kilometers in zones 3–4, kilometers in zones 5–T1–T2, sprint kilometers, strength-training frequency, and hours of alternative training. Internal-response indicators were perceived exertion, perceived training success, and perceived recovery. These internal variables are proxies for athlete response; they are not validated latent constructs and are not treated as reflective scales. Consequently, SEM, CFA, mediation, and reliability coefficients are inappropriate.

No mediators or moderators were specified because the dataset lacks variables and temporal measurement needed to identify mediation or moderation credibly. Athlete identifier was used as the grouping variable for validation, not as a conventional fixed-effect predictor. Date was retained for record tracing but not entered as a raw predictor, thereby avoiding a model that might learn calendar position. Age, sex, event specialty, prior injury, biomechanics, sleep, nutrition, mental stress, menstrual health, treatment, and environmental exposure were unavailable. Their absence is a source of omitted predictive information, not a basis for imputation or invention. Table 2 summarizes the predictor streams, measurement types, and model handling.

**Table 2:** Predictor organization in the seven-day daily sequence.

Stream	Variable	Type	Model handling
External	Number of sessions	Count	Daily; summed descriptively
External	Total kilometers	Distance	Daily; summed
External	Kilometers in zones 3–4	Distance	Moderate-intensity proxy
External	Kilometers in zones 5–T1–T2	Distance	Higher-intensity proxy
External	Sprint kilometers	Distance	High-speed exposure proxy
External	Strength training	Count	Session/frequency indicator
External	Hours alternative training	Hours	Cross-training exposure
Internal	Perceived exertion	Normalized rating	Availability mask added
Internal	Perceived training success	Normalized rating	Availability mask added
Internal	Perceived recovery	Normalized rating	Availability mask added

Each variable was ordered from Day −7 to Day −1. The −0.01 sentinel in subjective variables was treated as unavailable on zero-session days, not as a negative rating.

### 3.3 Data audit and preprocessing

There were no blank cells and no duplicate athlete-date rows in the daily data set. But, there were 241,047 cells with the sentinel value of −0.01. These values were observed during the zero session days in the subjective variables during inspection. If they were to be treated as normal negative ratings, an artificial rating scale would be created and if they were to be deleted then rest days would be lost and the temporal process would be modified. Analysis thus employed a neutral numeric value for the sentinel values which were fold-specifically scaled, and binary availability masks for each subjective variable and day. The masks enabled the model to differentiate between an unavailable rating and an actual observed rating.

Each training fold was used for all transformations. Training-fold medians and interquartile ranges were used to robustly scale the continuous inputs to minimize the impact of distance variables with non-normal distributions and extreme values. The resulting scaling parameters were then used with validation and test athletes. There was no oversampling or synthetic sequence generation of validation or test data. This was to prevent the synthetic examples from introducing temporal dependence or minority-class structure.

Seven-day aggregated variables were used to calculate descriptive statistics. External loads were added over the entire 7 day period. Internal-response means were computed using all of the ratings except the −0.01 sentinel. The interpretations of these summaries were limited to description of the cohort only and were not meant to be the basis for a causal comparison as more than one row per athlete and windows overlapped. For descriptive reporting, seven-day summaries were first averaged within each athlete and outcome-label category. The resulting athlete-level means were then summarized across athletes. Standard deviations and interquartile ranges in Table 3 therefore describe variation across athletes rather than across 42,766 overlapping windows. These summaries remain descriptive and are not independent causal comparisons.

**Table 3:** Athlete-level seven-day descriptive summaries by injury-label category.

Variable	No injury, n athletes	No injury mean (SD)	No injury median [IQR]	Injury-labeled, n athletes	Injury-labeled mean (SD)	Injury-labeled median [IQR]
Sessions	74	5.499 (1.555)	5.610 [4.641, 6.274]	63	6.185 (1.466)	6.286 [5.750, 6.952]
Total km	74	45.990 (25.763)	42.517[28.588,61.267]	63	54.893 (24.492)	51.388 [39.949,68.361]
Km zones 3–4	74	4.731 (4.092)	3.677 [1.993, 6.363]	63	6.697 (5.668)	4.909 [2.596, 9.700]
Km zones 5–T1–T2	74	3.531 (3.085)	3.330 [0.833,5.014]	63	4.287 (3.953)	3.500 [0.943, 6.922]
Sprint km	74	0.637 (1.936)	0.323 [0.000, 0.581]	63	1.266 (5.156)	0.300 [0.000, 0.775]
Strength sessions	74	0.748 (0.520)	0.807 [0.323, 1.062]	63	0.887 (0.625)	1.045 [0.275, 1.275]
Alternative-training hours	74	1.029 (1.108)	0.665 [0.119, 1.697]	63	0.991 (1.489)	0.643 [0.000, 1.405]
Perceived exertion	74	0.307 (0.152)	0.312 [0.158, 0.434]	62	0.346 (0.154)	0.364 [0.225, 0.459]
Perceived training success	74	0.408 (0.298)	0.480 [0.049, 0.664]	62	0.462 (0.271)	0.561 [0.241, 0.673]
Perceived recovery	74	0.247 (0.110)	0.240 [0.167, 0.308]	62	0.277 (0.112)	0.283 [0.175, 0.347]

Seven-day summaries were first averaged within each athlete and outcome-label category and were then summarized across athletes. External variables represent seven-day sums, whereas internal-response variables represent means over observed ratings. The injury-labeled subjective-rating summaries include 62 athletes because one injury-contributing athlete did not have usable observed ratings. The values are descriptive and should not be interpreted as independent causal effects.

### 3.4 Proposed DS-MTCN-IG model

The proposed Dual-Stream Multi-Scale Temporal Convolutional Network with Integrated Gradients explanation used separate temporal encoders for external-load and internal-response sequences. Causal convolutions preserved temporal order from Day −7 to Day −1. Parallel kernel sizes of 2, 3, and 5 allowed the network to represent short-range patterns across the seven-day window. The architecture was intentionally compact because the effective independent sample was 74 athletes rather than 42,766 independent participants.

Let $X_{E}\in {\mathbb{R}}^{7\times 7}$ denote the external-load sequence containing seven variables across 7 days, and let $X_{I}\in {\mathbb{R}}^{7\times 6}$ denote the internal-response sequence containing three measured ratings and three availability masks. For implementation, each tensor was transposed to channels-by-time form before entering the convolutional branches.

For each stream s∈{E,I} and kernel size k∈{2,3,5} , a branch contained two plain causal one-dimensional convolutional layers. Each layer used eight output channels, stride 1, dilation 1, kernel-specific padding of k−1 , right-side trimming to preserve causality and sequence length, GELU activation, and dropout 0.25. Residual blocks were not used. The final temporal output of each branch was:

$$h_{s,k}=\mathrm{TC}N_{s,k}{\left(X_{s}\right)}_{t=7},h_{s,k}\in {\mathbb{R}}^{8}$$

The three branch outputs were concatenated and projected:

$$H_{s}=\text{GELU}(W_{s}[h_{s,2};h_{s,3};h_{s,5}]+b_{s})$$

Where $[h_{s,2};h_{s,3};h_{s,5}]\in {\mathbb{R}}^{24}$ , $W_{s}\in {\mathbb{R}}^{16\times 24}$ , and $H_{E},H_{I}\in {\mathbb{R}}^{16}$ .

The gate was a 16-dimensional vector rather than a scalar:

$$g=\sigma (W_{g}[H_{E};H_{I}]+b_{g})$$

Where $W_{g}\in {\mathbb{R}}^{16\times 32}$ and $g\in {\mathbb{R}}^{16}$ . Fusion was performed element-wise:

$$H_{F}=g⊙H_{E}+(1-g)⊙H_{I},H_{F}\in {\mathbb{R}}^{16}$$

The notation $|H_{E}-H_{I}|$ denotes element-wise absolute difference, not a vector norm. The classifier input was:

$$Z=[H_{F};|H_{E}-H_{I}|H_{E}⊙H_{I}],Z\in {\mathbb{R}}^{48}$$

The classifier mapped 48→16→1 , with GELU and dropout 0.25 between the linear layers, followed by a sigmoid transformation for the reported probability. The full gated architecture contained 4,545 trainable parameters. The ungated dual-stream, internal-only, and external-only variants contained 3,761, 1,857, and 1,937 trainable parameters, respectively.

### 3.5 Class imbalance, optimization, calibration, and reproducibility

Class-balanced focal loss was used to reduce domination by easy non-injury observations. For binary target y and predicted probability p, the focal loss is:

FL(pt)=−αt(1−pt)^γlog(pt).

where pt. is p for injury observations and 1 − p otherwise, αt weights class frequency, and γ controls the down-weighting of easy cases (34). The analysis used γ = 2 and class-balanced weighting estimated within each training fold. Models were optimized with AdamW using learning rate 0.001 and decoupled weight decay 0.0001 (35). Gradient norms were clipped at 5.0. ReduceLROnPlateau monitored validation PR-AUC, used reduction factor 0.5 and patience 2, and reduced the learning rate after two validation plateaus. Training was limited to eight epochs, and early stopping retained the epoch with the highest validation PR-AUC after three epochs without improvement. The architecture used eight hidden channels per temporal branch, a 16-unit fusion representation, dropout 0.25, and batches of 2,048 observations.

Post-hoc Platt calibration was fitted separately within each outer-fold development set using validation-set predictions and was applied to the corresponding outer-test probabilities for DS-MTCN-IG, logistic regression, and XGBoost. No outer-test outcomes were used to estimate the calibration models. ROC-AUC, PR-AUC, and Brier score were designated as the primary cross-model measures because they do not depend on a classification threshold. For a descriptive and directly comparable operating-point analysis, the same fixed threshold of 0.013632, corresponding to the source-cohort injury-label prevalence, was applied to the out-of-fold probabilities from all three models. This common threshold was not interpreted as a clinical cut-off or optimized decision rule. The full gated model contained 4,545 trainable parameters. The ungated dual-stream, internal-only, and external-only models contained 3,761, 1,857, and 1,937 parameters, respectively. In the reproducibility rerun, training was performed on an AMD EPYC 9 V74 CPU using PyTorch 2.10 in CPU mode with four processing threads. Mean gated-model training time was 3.91 s per outer fold and seed (SD 0.66 s), excluding data loading, attribution computation, and aggregate post-processing. These runtime values describe the reported reproducibility environment and are not hardware-independent benchmarks.

### 3.6 Baselines, ablations, and validation

Logistic regression was included as an interpretable linear baseline and XGBoost as a nonlinear tree-based baseline. Both used the same athlete-disjoint folds and fold-specific preprocessing. The deep architecture was evaluated against three ablations: an external-load branch only, an internal-response branch only, and dual streams concatenated without the learned gate. The prespecified primary neural-network seed was 42. Robustness analysis repeated the full architecture and all three ablations with seeds 123, 999, 2024, and 5,678 while holding the outer and inner athlete splits constant, thereby isolating stochastic training variation from split variation. Logistic regression used an L2 penalty, C=1.0 , the liblinear solver, a maximum of 2,000 iterations, balanced class weights, and random state 42. XGBoost used 300 trees, maximum depth 3, learning rate 0.03, subsample 0.80, column subsampling 0.80, binary logistic objective, log-loss evaluation, random state 42, and four processing threads. The positive-class weight was calculated separately within each training fold as $N_{\text{negative}}/N_{\text{positive}}$ . Hyperparameters were fixed before outer-fold evaluation and were not selected using outer test data.

Outer validation used StratifiedGroupKFold(n_splits = 5, shuffle = True, random_state = 42), with the binary injury label supplied for approximate stratification and athlete identifier supplied as the grouping variable. All observations from an athlete were assigned to one and only one outer test fold. Within each outer development set, a separately instantiated five-fold StratifiedGroupKFold generated a grouped validation partition; the first inner split was used for validation, while the remaining development athletes were used for model fitting. The validation partition was used for early stopping, probability calibration, and the original training workflow, without access to the outer test outcomes.

Approximate balancing was achieved at the athlete level rather than by distributing individual injury-labeled rows directly. The resulting outer folds contained 14–16 athletes, 8,506–8,593 athlete-day rows, and 115–118 injury-labeled rows, with prevalence ranging from 0.01346 to 0.01387. Complete per-fold composition is reported in Supplementary Table S1.

**Figure 2:**
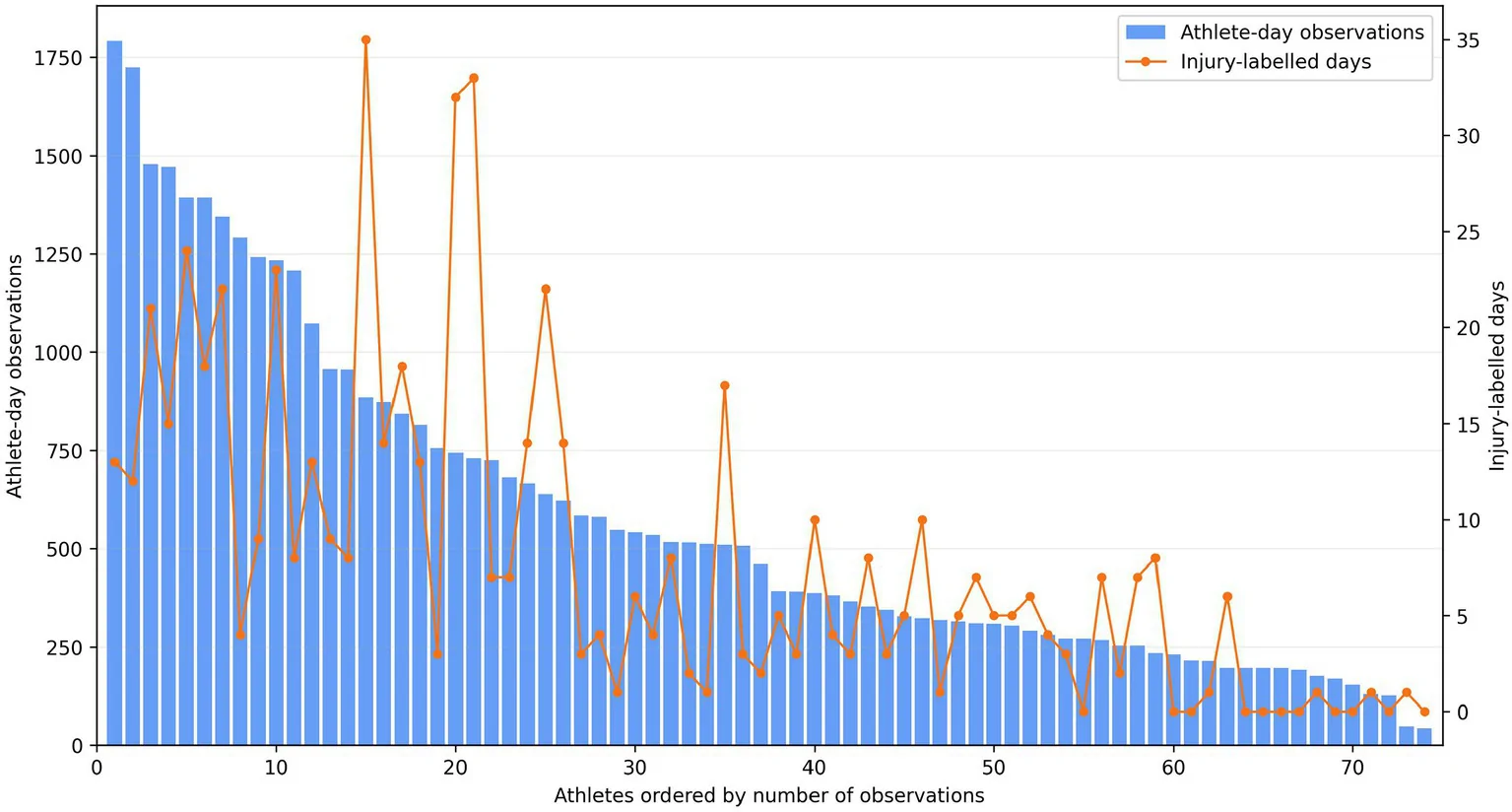
Unequal athlete follow-up and sparse injury-labeled days. Bars show athlete-day observations using the left vertical axis, and the line shows injury-labeled days using the right vertical axis. Injury counts were not multiplied or otherwise rescaled.

This is an evaluation of transportability that is not dependent on the athlete. It is more stringent than row-random split and is different from personalized forecasting design. As shown in Figure 2, there is also significant difference in the length of follow-up and injuries between athletes, further emphasizing the need for group-aware analysis. The range of observations made by the smallest athlete was 43 and the largest was 1,791, while the range of counts for injuries was 0 to 35. Consecutive prediction records were not statistically independent because adjacent targets shared six of their seven historical days. The injury label itself was not entered as a predictor, so this was not direct feature-level target leakage. Nevertheless, Day −1 training and availability inputs for a target could reflect behavior occurring immediately after a preceding injury-labeled day, and adjacent windows represented highly similar exposure histories. This creates temporal adjacency dependence and possible outcome-proximity information. The number of independent clustering units was therefore 74 athletes rather than 42,766 independent observations.

A non-overlapping-target sensitivity analysis was conducted using seven Date-modulo-7 offsets. Within each offset, successive target dates for an athlete were separated by at least 7 days. The model-development, validation, and test records were each restricted to the corresponding offset, and every model was refitted under the same athlete-disjoint outer folds. Results were summarized across all seven offsets rather than selecting the best-performing offset.

### 3.7 Performance metrics, uncertainty, and explanations

ROC-AUC measured rank discrimination across thresholds. PR-AUC, implemented as average precision, was emphasized because event prevalence was 1.36% (17). Sensitivity, specificity, precision, F1, balanced accuracy, Matthews correlation coefficient, and confusion-matrix counts were calculated using the same fixed reporting threshold of 0.013632 for all three models. Brier score assessed probability error. The primary run reports pooled out-of-fold results and fold variation; the five-seed sensitivity analysis reports mean and standard deviation across seed-level fold means.

For the proposed model, athlete-cluster bootstrap resampling was used to estimate 95% confidence intervals for ROC-AUC, PR-AUC, and Brier score. Resampling at the athlete level preserves within-athlete dependence better than resampling individual rows. Pairwise model comparisons were performed using a paired athlete-cluster bootstrap. The 74 athletes were sampled with replacement for 2,000 bootstrap replicates, and all observations belonging to each sampled athlete were retained together. For each replicate, differences in ROC-AUC and Brier score were calculated between models using predictions from the same resampled athletes. Two-sided *p* values were estimated from the proportions of bootstrap differences on either side of zero, and percentile 95% confidence intervals were reported. Athlete-cluster bootstrap inference was preferred to a conventional row-level DeLong test because the athlete-day observations and overlapping windows were not independent.

For the non-overlapping analysis, the seven Date-modulo-7 offsets were treated as paired evaluation conditions because each model was fitted and evaluated on the same offset-specific records. Pairwise ROC-AUC differences were assessed using exact two-sided Wilcoxon signed-rank tests across the seven offset-level values. The analysis focused primarily on logistic regression vs. DS-MTCN-IG. Comparisons involving XGBoost were treated as exploratory. Because only seven offsets were available and their injury-label prevalence and positive-event counts differed, these tests were interpreted cautiously and were not treated as substitutes for prediction-level or external validation.

**Figure 3:**
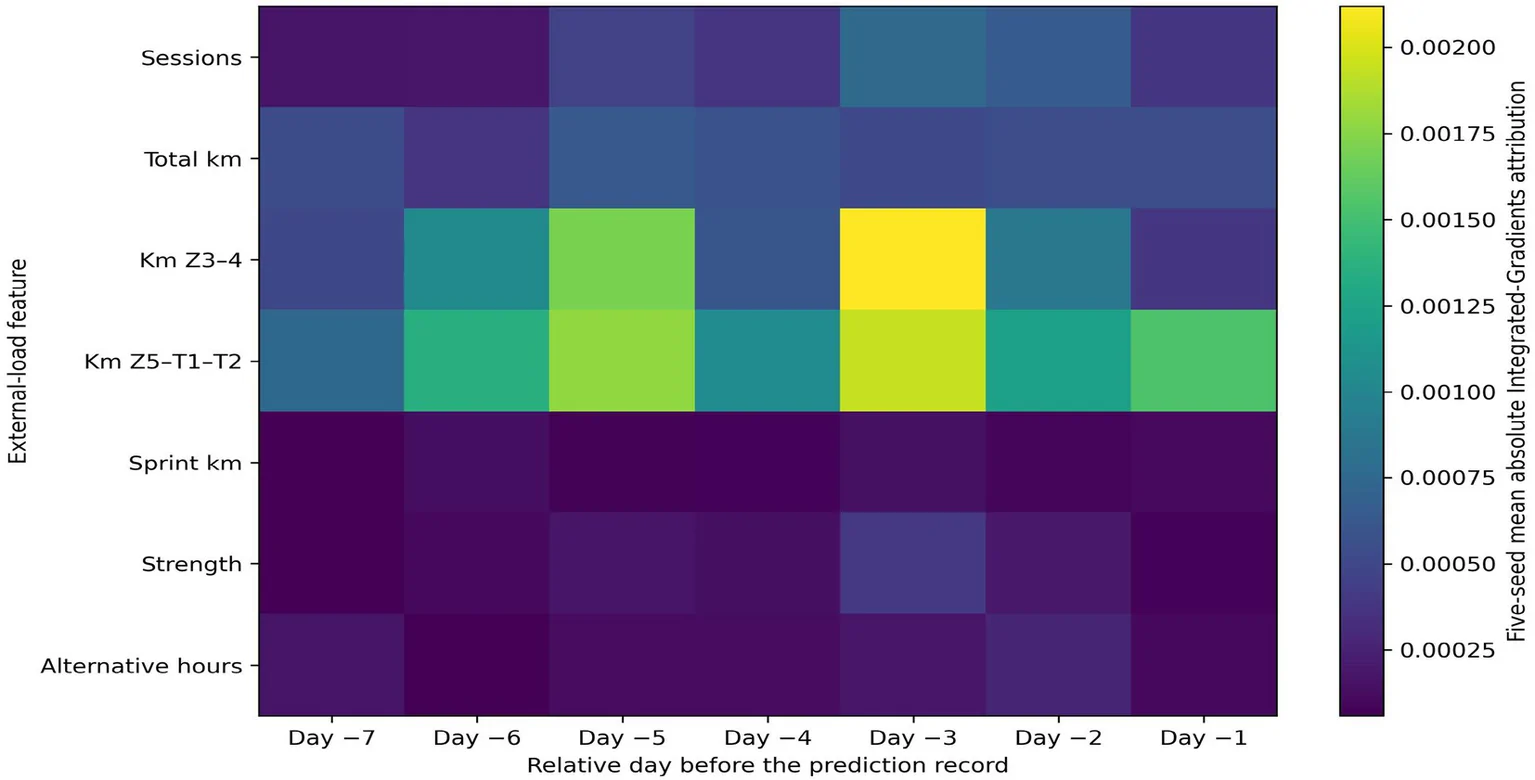
External-load feature-by-day Integrated-Gradients attribution. Values are five-seed mean absolute Integrated-Gradients attributions calculated from the deterministic subset of 100 injury-labeled held-out records per seed.

**Figure 4:**
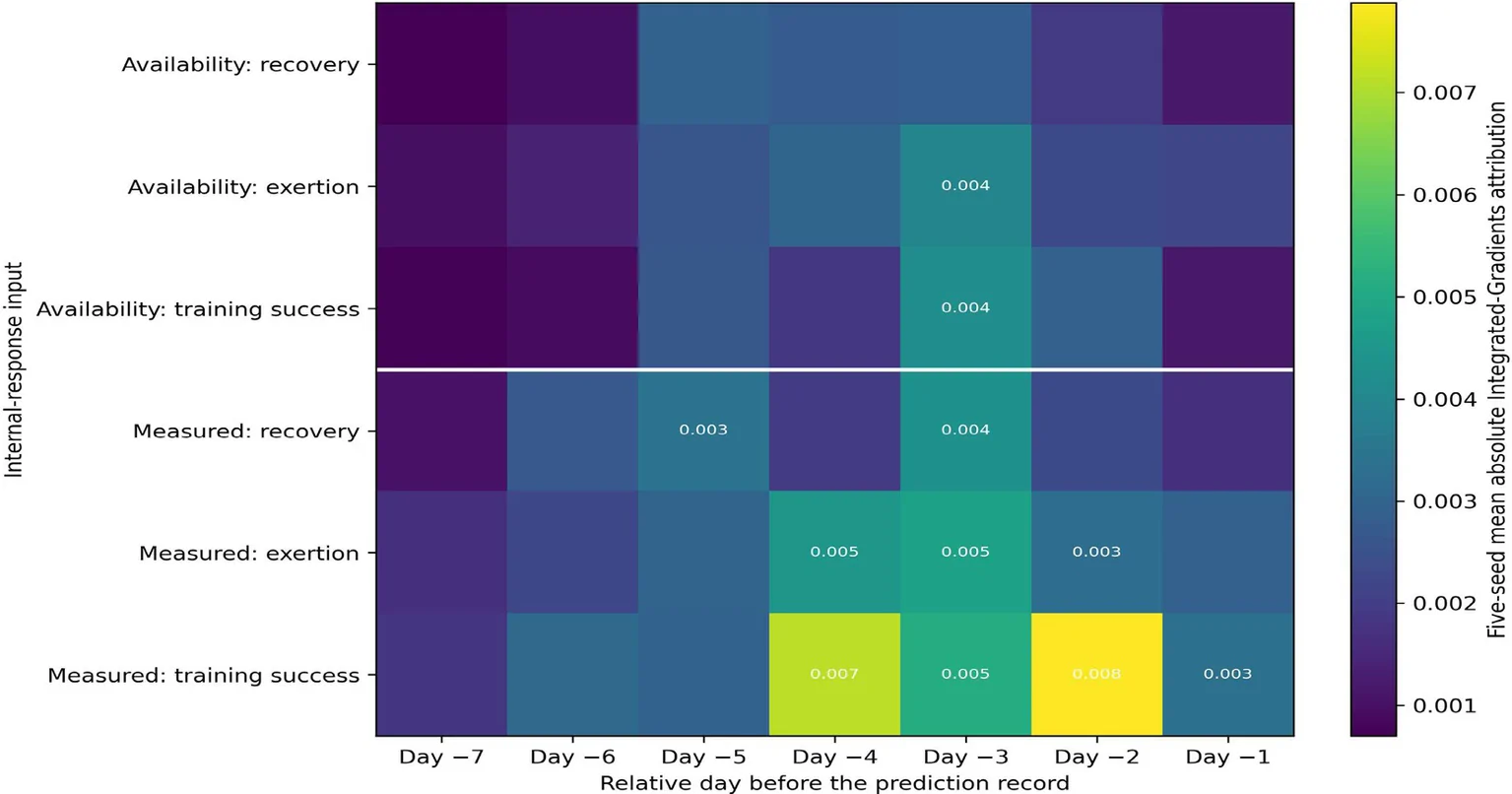
Internal-response attribution map separating measured ratings from availability masks. Values are five-seed mean absolute Integrated-Gradients attributions calculated from the deterministic subset of 100 injury-labeled held-out records per seed. Availability-mask rows indicate whether subjective ratings were observed. High attribution to these rows indicates model reliance on rating availability, which may reflect recording behavior, genuine rest-day or training-participation structure, or post-injury recovery and outcome-proximity information. It should not be interpreted as a physiological injury mechanism or intervention target.

Integrated Gradients were recomputed for all five neural-network seeds using 64 trapezoidal integration steps and model logits as the explained output. The zero baseline in robustly scaled space corresponds to the training-fold median for measured continuous variables; it therefore does not represent zero training load or a physiological rest day. For availability masks, zero represents an unavailable rating. This baseline distinction was retained explicitly when interpreting the attributions. Completeness was evaluated for every explained record by comparing the sum of the Integrated-Gradients attributions with the logit difference $F(x)-F(x^{\prime })$ . The median absolute completeness error was $3.94\times 10^{-5}$ , the 95th-percentile absolute error was $3.60\times 10^{-4}$ , and the median relative error was $9.25\times 10^{-4}$ . Explanations were aggregated separately for each seed before calculating five-seed means, between-seed standard deviations, ranks, and rank-stability statistics. Quantitative calibration assessment included calibration intercept, calibration slope, and E50. Calibration intercept and slope were estimated by regressing the observed binary outcome on the logit of the pooled out-of-fold probability; ideal values are 0 and 1, respectively. E50 was defined as the median absolute difference between observed event frequency and mean predicted probability across 10 equal-frequency probability bins. Calibration curves were treated as descriptive because of the small number of independent athletes and the rare outcome. For computationally standardized attribution analysis, each outer fold contributed a deterministic subset consisting of the first 20 injury-labeled and first 20 non-injury held-out records. Attribution summaries reported in Table 4 and Figures 3, 4 were calculated from the injury-labeled subset, corresponding to 100 held-out injury-labeled records per seed.

**Table 4:** Descriptive five-seed integrated-gradients attribution summaries for the deterministic injury-labeled held-out subset.

Stream	Relative day	Input type	Feature	Five-seed mean |IG|	Between-seed SD	Median rank	Rank range
Internal	Day −2	Measured rating	Perceived training success	0.007877	0.004115	1	1–11
Internal	Day −4	Measured rating	Perceived training success	0.007170	0.003677	2	1–17
Internal	Day −3	Measured rating	Perceived training success	0.005157	0.002693	7	3–25
Internal	Day −3	Measured rating	Perceived exertion	0.004812	0.003248	10	2–26
Internal	Day −4	Measured rating	Perceived exertion	0.004505	0.001977	8	4–26
Internal	Day −3	Measured rating	Perceived recovery	0.004311	0.000898	7	5–11
Internal	Day −3	Availability mask	Perceived training success available	0.004231	0.002370	11	2–26
Internal	Day −3	Availability mask	Perceived exertion available	0.003956	0.002270	10	3–38
Internal	Day −5	Measured rating	Perceived recovery	0.003437	0.001538	9	7–41
Internal	Day −1	Measured rating	Perceived training success	0.003359	0.001716	18	6–25
Internal	Day −2	Measured rating	Perceived exertion	0.003261	0.001744	16	6–33
Internal	Day −6	Measured rating	Perceived training success	0.003168	0.001901	15	5–35
Internal	Day −4	Availability mask	Perceived exertion available	0.003030	0.001191	19	3–34
Internal	Day −5	Measured rating	Perceived exertion	0.002990	0.000606	16	11–18
Internal	Day −5	Measured rating	Perceived training success	0.002965	0.001939	17	5–27
Internal	Day −5	Availability mask	Perceived recovery available	0.002962	0.001818	12	6–33
Internal	Day −2	Availability mask	Perceived training success available	0.002892	0.001845	27	5–30
Internal	Day −1	Measured rating	Perceived exertion	0.002889	0.002145	14	7–54
Internal	Day −3	Availability mask	Perceived recovery available	0.002857	0.001718	17	4–46
Internal	Day −4	Availability mask	Perceived recovery available	0.002797	0.001652	24	9–41

Values are descriptive five-seed summaries of absolute Integrated-Gradients attribution calculated from a deterministic subset of 100 injury-labeled held-out records per seed, consisting of up to 20 injury-labeled records from each outer test fold. They should not be used to identify definitive or model-independent “most important” features. Complete-ranking Spearman correlations ranged from 0.790 to 0.881, whereas pairwise overlap among the 10 highest-ranked inputs ranged from 2 to 7 features, corresponding to Jaccard indices of 0.111 to 0.538. Several individual features also had wide rank ranges across seeds. The displayed ordering therefore summarizes average model reliance within the explained subset and does not establish a stable feature hierarchy, causal importance, or an intervention target.

### 3.8 Ethics, reproducibility, and reporting

The analysis used publicly available anonymized secondary data and did not recruit participants, contact athletes, or access direct identifiers. No new institutional ethics approval was required for the secondary analysis. The ethical status and consent procedures of the original data collection remain those reported by the source investigators.

The analytical workflow produced fold-level predictions, confusion matrices, saved model weights, calibration outputs, ablation results, multi-seed sensitivity results, and feature-attribution tables. The primary seed and four sensitivity seeds were prespecified in the computational workflow. Reporting was guided by TRIPOD+AI and appraised against PROBAST+AI principles (32, 33). Because this was a retrospective secondary-data study without prospective decision use, the model is described as a research prediction model rather than a clinical or coaching decision rule. Accordingly, no output from the model is interpreted as an alert, intervention trigger, clinical diagnosis, or athlete-selection tool.

## 4 Results

### 4.1 Cohort and descriptive results

The daily analysis included 42,766 athlete-day records from 74 runners. There were 583 injury-labeled observations and 42,183 non-injury observations, corresponding to 1.363% prevalence. The imbalance was therefore approximately 72 non-injury observations per injury-labeled observation. Follow-up differed markedly between athletes, and several athletes had few or no injury-labeled observation.

At the athlete level, mean seven-day session count was 6.185 in injury-labeled categories and 5.499 in no-injury categories. Corresponding mean total distance was 54.893 vs. 45.990 km, moderate-intensity distance was 6.697 vs. 4.731 km, higher-intensity distance was 4.287 vs. 3.531 km, and sprint distance was 1.266 vs. 0.637 km. Mean perceived exertion, training success, and recovery were also descriptively higher in injury-labeled categories. These summaries were calculated after aggregation within athlete and outcome-label category and should not be interpreted as independent or causal comparisons.

### 4.2 Main predictive performance

Table 5 reports the revised pooled out-of-fold performance. DS-MTCN-IG achieved ROC-AUC 0.596, PR-AUC 0.0204, and Brier score 0.01343. Logistic regression achieved ROC-AUC 0.619, PR-AUC 0.0197, and Brier score 0.01356, whereas XGBoost achieved ROC-AUC 0.580, PR-AUC 0.0181, and Brier score 0.01343. All PR-AUC values were only slightly above the injury-label prevalence of 0.0136. Pairwise athlete-cluster bootstrap analysis showed that the numerical ROC-AUC difference between logistic regression and DS-MTCN-IG was not statistically significant (*Δ* = 0.0227, 95% CI − 0.0180 to 0.0714; *p* = 0.291). Logistic regression significantly exceeded XGBoost, whereas XGBoost and DS-MTCN-IG did not differ significantly.

**Table 5:** Athlete-independent pooled out-of-fold model performance in the revised primary analysis.

Model	ROC-AUC	PR-AUC	Brier score	Naïve Brier	Brier skill	Calibration intercept	Calibration slope	E50	Sensitivity	Specificity	Precision	F1	MCC	TP	FP	FN	TN
DS-MTCN-IG	0.596	0.0204	0.013427	0.013446	0.0015	−0.219	0.950	0.001445	0.611	0.533	0.0178	0.0345	0.0334	356	19,694	227	22,489
Logistic Regression	0.619	0.0197	0.013560	0.013446	−0.0084	−1.507	0.647	0.002119	0.599	0.582	0.0194	0.0376	0.0424	349	17,636	234	24,547
XGBoost	0.580	0.0181	0.013434	0.013446	0.0010	−0.219	0.948	0.001133	0.588	0.540	0.0174	0.0337	0.0298	343	19,414	240	22,769

All threshold-dependent measures were calculated using the same fixed threshold of 0.013632, corresponding to the empirical injury-label prevalence. This threshold was used only for comparable descriptive operating-point analysis and should not be interpreted as a clinical cut-off. PR-AUC was calculated as average precision. The naïve Brier score was obtained by assigning the empirical prevalence to every observation. Brier skill score was calculated relative to this prevalence-only model. Ideal calibration intercept and slope values are 0 and 1, respectively. E50 represents the median absolute calibration error across 10 equal-frequency probability groups.

Pairwise athlete-cluster bootstrap comparisons did not show a statistically significant ROC-AUC difference between logistic regression and DS-MTCN-IG (Δ = 0.0227, 95% CI − 0.0180 to 0.0714; *p* = 0.291). Logistic regression had a statistically higher ROC-AUC than XGBoost (Δ = 0.0383, 95% CI 0.0087 to 0.0697; *p* = 0.012). The ROC-AUC difference between XGBoost and DS-MTCN-IG was not statistically significant (Δ = −0.0155, 95% CI − 0.0507 to 0.0268; *p* = 0.409). None of the pairwise Brier-score differences was statistically significant. Logistic regression should therefore be described as numerically, rather than conclusively, more discriminative than DS-MTCN-IG. A constant model assigning the empirical injury-label prevalence of 0.013632 to every observation produced a Brier score of 0.0134465. DS-MTCN-IG achieved Brier score 0.0134268 and Brier skill score 0.0015; XGBoost achieved 0.0134337 and Brier skill score 0.0010; logistic regression achieved 0.0135598 and Brier skill score −0.0084. Thus, the probability-error improvements of DS-MTCN-IG and XGBoost over the naïve prevalence model were negligible, while logistic regression was slightly worse. These results do not support useful individualized probability estimation despite modest rank discrimination.

The apparent contradiction between discrimination and probability accuracy is methodologically important. ROC-AUC evaluates ranking discrimination, whereas the Brier score and calibration parameters evaluate the accuracy of predicted probabilities. Logistic regression achieved the highest ROC-AUC (0.619), but after the same validation-only Platt-calibration procedure used for DS-MTCN-IG and XGBoost, its pooled out-of-fold probabilities remained substantially miscalibrated, with a calibration intercept of −1.507 and slope of 0.647. Its Brier skill score of −0.0084 therefore indicates slightly worse probability error than the constant-prevalence benchmark. DS-MTCN-IG and XGBoost showed lower discrimination but calibration intercepts closer to zero, slopes closer to one, and slightly positive Brier skill scores. Because the same validation-only calibration workflow was applied to all three models, these Brier-score differences cannot be attributed to differential use of post-hoc calibration. Rather, they demonstrate that ranking discrimination and absolute probability accuracy answer different questions and that fold-specific calibration did not fully correct pooled logistic-regression miscalibration. None of these probability estimates should be interpreted as clinically usable risk estimates.

**Figure 5:**
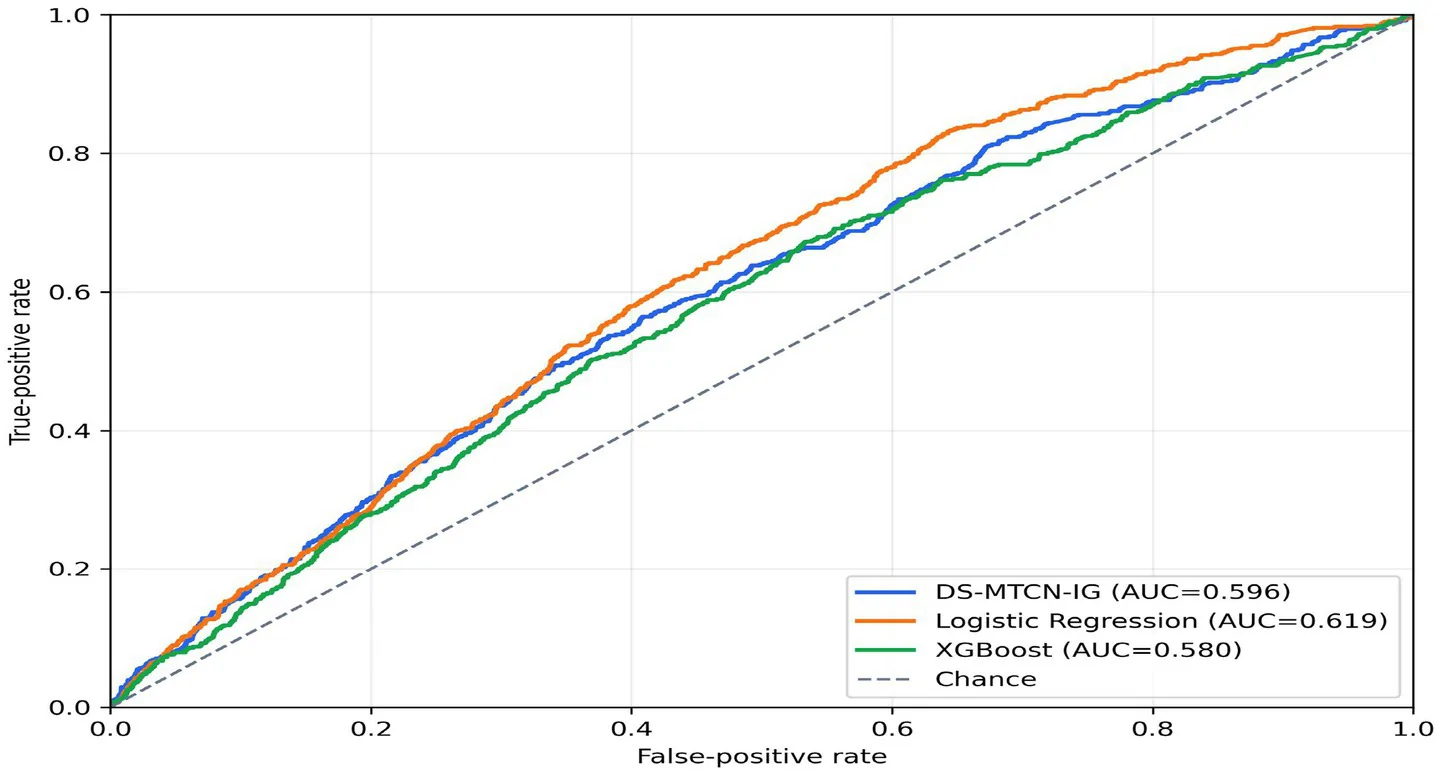
Athlete-independent out-of-fold receiver-operating-characteristic curves.

**Figure 6:**
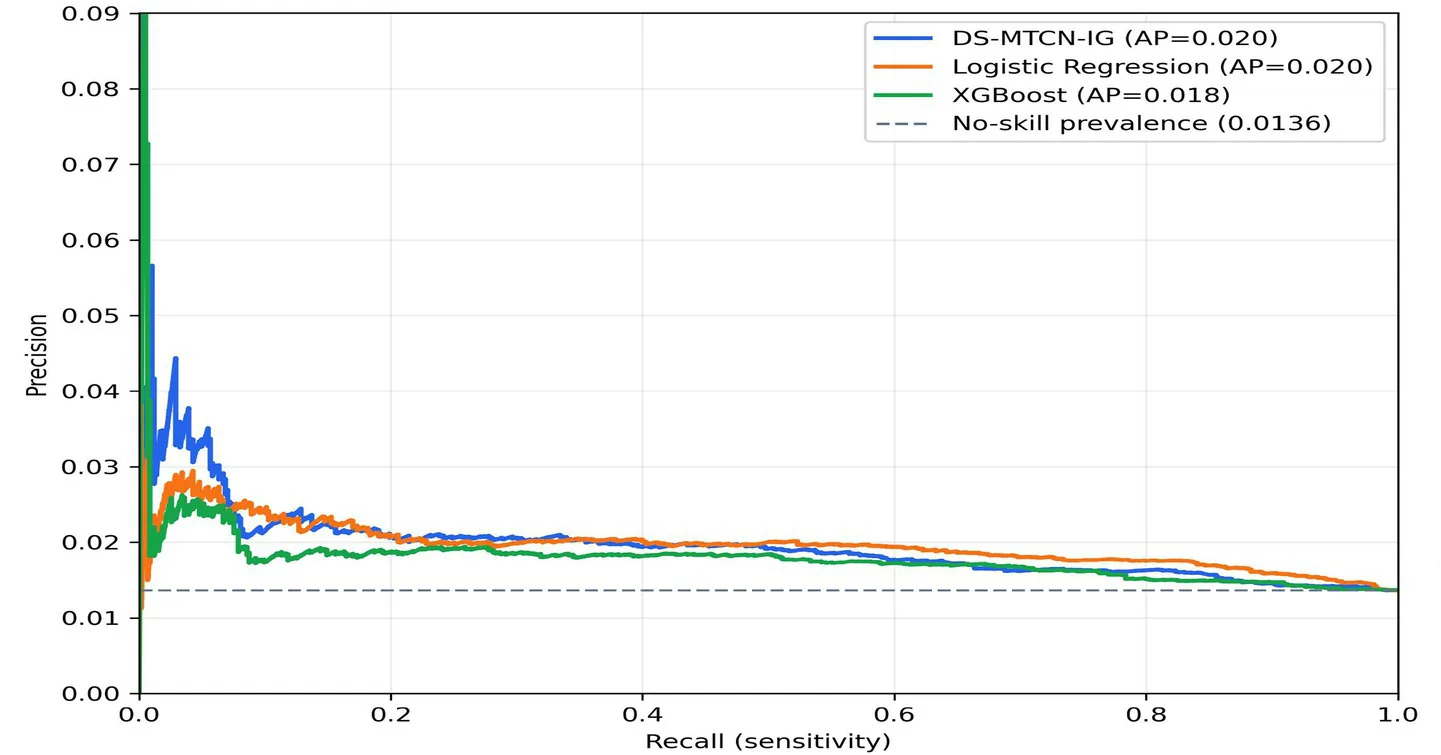
Athlete-independent precision–recall curves. The dashed horizontal line is the no-skill injury prevalence of 1.36%.

**Figure 7:**
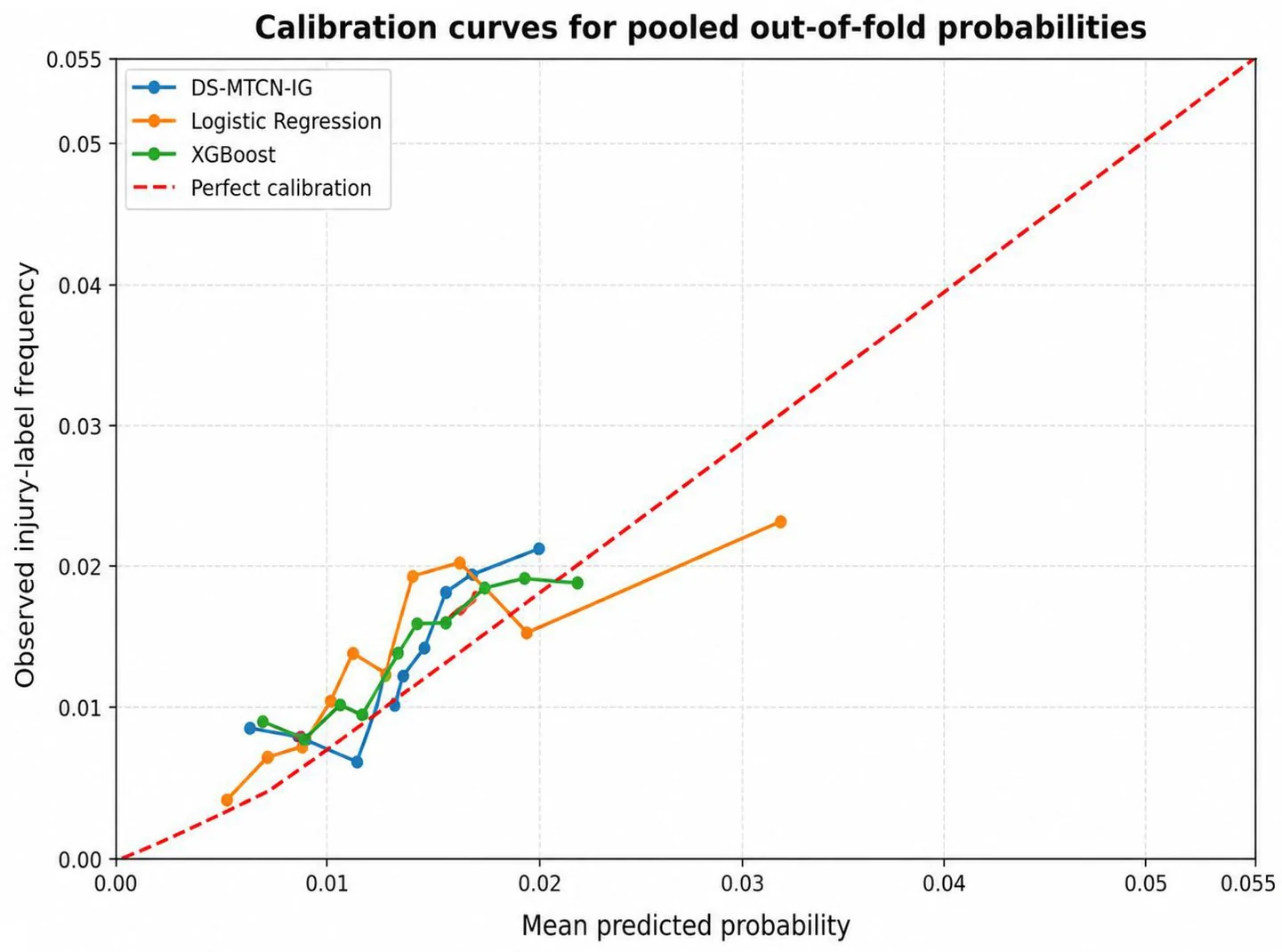
Calibration curves for pooled out-of-fold probabilities. Curves are restricted to the low-probability range relevant to the 1.36% injury-label prevalence. Calibration is presented descriptively because of the small number of independent athletes and the rare outcome and does not establish clinical utility.

At the common reporting threshold of 0.013632, DS-MTCN-IG achieved sensitivity 0.611, specificity 0.533, precision 0.0178, and MCC 0.0334. Logistic regression achieved sensitivity 0.599, specificity 0.582, precision 0.0194, and MCC 0.0424. XGBoost achieved sensitivity 0.588, specificity 0.540, precision 0.0174, and MCC 0.0298. The uniformly low precision and weak MCC values indicate that none of the models provided a practically decisive operating point. The common threshold was used solely for descriptive comparability and is not a clinical decision threshold. Figures 5–7 present the revised pooled ROC, precision–recall, and calibration results corresponding to the values reported in Table 5.

### 4.3 Sensitivity analysis using non-overlapping prediction targets

Performance weakened materially after temporal overlap was removed. Across the seven non-overlapping offsets, DS-MTCN-IG achieved mean ROC-AUC 0.469 (SD 0.028; range 0.429–0.514) and mean PR-AUC 0.0126 (SD 0.0039). Logistic regression achieved mean ROC-AUC 0.517 (SD 0.032; range 0.468–0.570), whereas XGBoost achieved mean ROC-AUC 0.493 (SD 0.056; range 0.434–0.602). The gated model therefore did not retain above-chance discrimination when adjacent overlapping targets were removed. This sensitivity result materially weakens the interpretation that the full-window analysis identified a stable athlete-independent temporal signal. Interpretation across offsets is limited by substantial heterogeneity in injury-label prevalence and positive-event counts. Prevalence ranged from 1.03 to 2.33%, with 63 to 143 injury-labeled observations per offset. The observed ROC-AUC variation may therefore reflect differences in case composition and estimation precision as well as model instability. The mean offset-level performance values should be interpreted as descriptive summaries rather than as estimates from seven equivalent prediction tasks. In the paired offset-level analysis, logistic regression exceeded DS-MTCN-IG by a mean ROC-AUC difference of 0.048 and a median paired difference of 0.043; the exact two-sided Wilcoxon signed-rank test was statistically significant (*p* = 0.031). Logistic regression exceeded XGBoost by a mean difference of 0.024 and a median difference of 0.019, but this comparison was not statistically significant (*p* = 0.469). XGBoost exceeded DS-MTCN-IG by a mean difference of 0.025 and a median difference of 0.015, which was also not statistically significant (*p* = 0.297). These results provide additional evidence that the gated deep model offered no predictive advantage under the non-overlapping design. Given only seven paired offsets and the offset-specific prevalence range of 1.03–2.33%, the *p* = 0.031 result is statistically fragile and may partly reflect prevalence-related differences in case composition, task difficulty, and estimation precision rather than a stable model effect. It should therefore be interpreted as supportive rather than definitive evidence of a model difference.

### 4.4 Ablation and fusion results

Across five seeds, the ungated dual-stream model achieved the highest mean ROC-AUC among the deep variants (0.604 ± 0.011), followed by the full gated model (0.598 ± 0.007), internal-only model (0.575 ± 0.029), and external-only model (0.538 ± 0.021). The gated architecture therefore did not improve discrimination over simple concatenation, and H2 was rejected. The mean external-stream gate values remained close to 0.5 across held-out folds and seeds. A value near 0.5 indicates that the learned fusion behaved largely as near-uniform averaging rather than strong feature-wise selection of one stream over the other. The gate did not collapse completely to a single stream, but its near-balanced values and absence of incremental performance do not support a claim of effective selective fusion (Table 6).

**Table 6:** Five-seed robustness and ablation analysis under athlete-independent grouped five-fold cross-validation.

Architecture	Seeds	ROC-AUC mean ± SD	ROC-AUC range	PR-AUC mean ± SD	Sensitivity mean ± SD	Specificity mean ± SD	MCC mean ± SD
DS-MTCN-IG	5	0.598 ± 0.007	0.586–0.604	0.0194 ± 0.0010	0.528 ± 0.089	0.603 ± 0.067	0.031 ± 0.006
Dual stream without gate	5	0.604 ± 0.011	0.584–0.613	0.0199 ± 0.0007	0.556 ± 0.034	0.591 ± 0.047	0.035 ± 0.007
Internal only	5	0.575 ± 0.029	0.526–0.595	0.0185 ± 0.0018	0.548 ± 0.155	0.558 ± 0.116	0.025 ± 0.011
External only	5	0.538 ± 0.021	0.512–0.569	0.0157 ± 0.0009	0.177 ± 0.098	0.862 ± 0.074	0.012 ± 0.007

Results summarize the five prespecified seeds 42, 123, 999, 2024, and 5,678. Complete athletes, rather than individual windows, were assigned to the outer test folds. Sensitivity, specificity, and MCC were calculated using the common prevalence threshold of 0.013632. The ungated dual-stream model produced the highest five-seed mean ROC-AUC among the deep-learning variants; consequently, the gated-fusion hypothesis was not supported.

### 4.5 Explainability results

Across the five neural-network seeds, measured internal-response ratings and availability masks both contributed to the fitted scores. The highest five-seed mean absolute attributions were observed for perceived training success on Days −2 and −4, while availability indicators for training success, exertion, and recovery also appeared among highly ranked inputs. Pairwise Spearman correlations of the complete attribution rankings ranged from 0.790 to 0.881, indicating moderately consistent broad ranking structure. However, only two to seven of the 10 highest-ranked inputs overlapped between individual seed pairs, showing that exact top-input rankings were seed-sensitive.

Availability-mask attribution has at least three plausible interpretations. First, it may represent recording behavior, questionnaire completion, or platform-specific monitoring practice. Second, because sentinel values occurred predominantly on zero-session days, the masks may encode legitimate training participation and planned rest-day structure. Third, reverse causality or outcome-proximity information may be present. If an injury episode began before or continued across the labeled target day, zero-session or post-injury rest days could enter the preceding seven-day predictor window. In that situation, the mask could help classify an injury-labeled day because the injury had already changed training participation or questionnaire availability, rather than because the mask predicted a future injury. The present dataset does not provide sufficiently precise onset, duration, recovery, diagnosis, or time-loss information to distinguish recording artifact, legitimate rest-day structure, and post-injury behavioral change. Availability-mask attribution is therefore treated as an observation-process audit and cannot be interpreted as a physiological mechanism, a prospective risk factor, or evidence of target leakage from the label itself.

### 4.6 Hypothesis summary

H1 was not supported in the non-overlapping evaluation, which is treated as the primary interpretation. Although ROC-AUC and PR-AUC exceeded their no-skill references in the overlapping-window analysis, that apparent support disappeared when temporal adjacency between successive targets was removed. H2 was not supported. The ungated dual-stream model produced the highest five-seed mean ROC-AUC among the deep variants, and the learned gate did not provide robust incremental discrimination. The gate should therefore be treated as a negative architectural finding. H3 was supported only as a statement about model reliance: external and internal streams both contributed. The prominence of availability masks limits physiological interpretation and confirms that attribution is an audit tool rather than causal evidence.

## 5 Discussion, contributions, and implications

### 5.1 Interpretation of the main findings

The most important finding was the failure of the gated model to retain above-chance discrimination after temporal adjacency was removed. Across the seven non-overlapping offsets, DS-MTCN-IG achieved a mean ROC-AUC of 0.469, compared with 0.596 in the full-window analysis. Because adjacent full-window observations shared six of seven historical days, the higher full-window estimate may reflect temporal autocorrelation, repeated exposure patterns, or outcome-proximity information rather than a stable predictive relationship between training-load history and injury-labeled days. The non-overlapping result should therefore be treated as the primary interpretive frame.

The proposed deep model consequently did not demonstrate a robust or practically useful predictive advantage. Logistic regression achieved the highest numerical ROC-AUC in the full-window analysis, but the paired athlete-cluster bootstrap difference from DS-MTCN-IG was not statistically significant. At the common reporting threshold, all models showed very low precision and weak Matthews correlation coefficients. These results do not support use of any model for athlete ranking, automated warning, training restriction, or clinical decision-making.

The defensible contribution of the deep architecture is methodological rather than operational. Its dual-stream structure enabled stream-specific ablation, temporal feature-by-day attribution, and examination of observation-availability patterns. These functions are useful for auditing how a temporal model uses longitudinal data, but they do not compensate for the non-overlapping null result, near-naïve probability accuracy, unstable exact attribution rankings, or the absence of clinically verified outcomes. On this dataset, model complexity was not justified by improved or robust predictive performance.

At the common reporting threshold, the three models showed broadly similar and weak operating characteristics. None achieved acceptable precision or MCC, and the dataset does not support selection of a clinically optimal threshold. Threshold-dependent differences should therefore be interpreted only as descriptive operating-point differences, not as evidence that one model offers greater clinical sensitivity or specificity.

It is worth emphasizing the PR-AUC values. Average precision of all three models was slightly higher than the 1.36% prevalence. Although the full-window ROC-AUC values were numerically above 0.5, the precision–recall curves demonstrate that positive predictions remained unreliable, and the apparent discrimination did not persist under the non-overlapping evaluation. This is an indication of why rare-event injury studies should not report accuracy or ROC-AUC alone. The model should not be used for athlete-level ranking or research triage because discrimination was weak and did not remain above chance in the non-overlapping-target sensitivity analysis. A decision-curve perspective further limits practical interpretation. With an injury-label prevalence of only 1.36% and ROC-AUC values near 0.60, the likely net benefit of threshold-based action would be small across most plausible decision thresholds unless the action were extremely low burden and low harm. The present study did not perform an intervention-simulation or decision-utility analysis, so the reported probabilities should not be used to trigger training restriction or medical intervention. Future studies should prespecify clinically meaningful actions, false-positive costs, false-negative costs, and decision thresholds before evaluating net benefit. Logistic regression’s higher ROC-AUC should not be interpreted as superior risk estimation because its negative Brier skill score and calibration intercept and slope showed that its probabilities were less accurate than the constant-prevalence benchmark.

### 5.2 Theoretical contribution

Dynamic load–capacity reasoning motivated the temporal organization of external-load and internal-response histories, but the present outcome and predictors do not provide a direct empirical test of that theory. The dataset does not observe tissue-specific load, tissue capacity, diagnosis, injury mechanism, pain onset, or severity. Consequently, neither discrimination nor feature attribution should be interpreted as confirmation of a biological load–injury pathway.

The architectural hypothesis concerning selective gated fusion was not supported. Across five seeds, the ungated dual-stream model achieved the highest mean ROC-AUC among the deep variants, while the learned gate remained close to uniform averaging and did not provide robust incremental discrimination. The gate is therefore best interpreted as an evaluated architectural mechanism that failed to demonstrate added value, rather than as an operational demonstration of dynamic load–response interaction.

The prominence of observation-availability masks adds a further theoretical insight. Athlete monitoring is a socio-technical process: what is recorded depends on schedules, compliance, platform design, and coaching practice. Prediction models can learn that process alongside athlete state. In this dataset, missing subjective ratings on zero-session days were structured rather than random. A theory of digital athlete monitoring should therefore include the measurement process as part of the system, not assume that the database is a transparent window onto physiology.

### 5.3 Methodological contribution

The evaluation framework is the best contribution. The athlete-level separation ensures that the model is not able to train on the same athlete’s baseline and test it. The consequences of rarity are apparent in the PR-AUC, MCC, Brier score, calibration curves, and the confusion-matrix counts. Five-seed sensitivity testing distinguishes between the two sources of training instabilities, stochastic and architecture comparison, and ablation and feature-by-day Integrated Gradients audit the value or use of component parts and temporal inputs. These elements address the shortcomings found in systematic reviews and in prediction-model advice (11, 32, 33).

The negative comparative result is also methodologically valuable. Many applied AI efforts involve choosing an advanced model, evaluating it in a manner that verifies its supposed superiority. In this case, logistic regression achieved a numerically higher ROC-AUC than DS-MTCN-IG, but the paired difference was not statistically significant. Reporting is a way of avoiding algorithmic solutionism, and providing reviewers with a more honest representation of what the data is able to support. Even though the deep model is valuable as a research object, its gated structure, and explanation layer shed light on the temporal information use, complexity does not mean practical utility.

Treatment of sentinel values is also important for reproducibility. If −0.01 is retained as an ordinary numeric value, it creates an artificial negative subjective rating. If it is simply replaced by a generic mean, the distinction between unavailable and observed ratings is erased. The masking approach preserved this information structure and made the mask effect visible in the attribution analysis. This result shows that missing-data handling is not a housekeeping detail but a modeling decision that can materially affect interpretation.

The attribution analysis does not establish that the model learned only recording-process leakage. Availability masks may encode both platform-dependent completion behavior and valid training-participation information, because subjective ratings were commonly unavailable on zero-session days. Reliance on availability indicators creates a portability concern if the masks primarily encode platform-specific completion behavior rather than genuine rest-day structure or post-injury recovery. A monitoring system requiring ratings every day, including rest and recovery days, could produce a different mask distribution and a different predictor–outcome relationship from the source dataset. Future studies should therefore compare full, masks-only, ratings-only, and no-mask models; use standardized reporting protocols; repeat explanations across seeds and alternative baselines; and test whether performance persists across platforms with different data-entry practices. Mandatory daily reporting may be evaluated as a study-design option, but it should not be presented as a conclusion directly established by the present attribution analysis.

### 5.4 Practical implications for coaches and sports-medicine teams

The model should be interpreted solely as a methodological research benchmark, not as a decision-support aid or automated injury-warning system. At the observed prevalence, positive classifications have low precision and should not directly restrict training, competition, selection, scholarship decisions, or medical clearance. Any review of a model-generated score would need to be combined with unmeasured clinical and contextual information, including pain, diagnosis, training phase, sleep, travel, surface, treatment, and athlete-reported concerns. The present dataset does not contain intervention outcomes or utility weights, so it cannot define a clinically optimal threshold.

The present findings do not empirically establish that any particular reporting protocol or injury definition will improve prediction. However, they identify design questions that future prospective studies should evaluate. These studies could compare daily reporting with session-only reporting, distinguish planned rest from non-completion, and use harmonized medical-attention, time-loss, and all-complaint injury definitions. Device specifications, training-zone definitions, and protocol changes could also be recorded prospectively. These are study-design recommendations for future evaluation rather than implications demonstrated by the current dataset.

In the future, wearable systems could provide the external stream with biomechanical and physiological data, but more data channels do not mean better prediction. There is some variation in the validity and field applicability of reviews on running wearables (8). New inputs should be added only after assessing their reliability and burden and their incremental value. It might be better to use a smaller, transparent system rather than one that is high dimensional and unmaintainable or unexplainable.

### 5.5 Speculative policy relevance for China

China is discussed only as a speculative, forward-looking policy context, not as an empirical application setting for the fitted model. The dataset was collected from a Dutch competitive running team and contains no Chinese participants, Chinese-language scale validation, Chinese injury-surveillance protocol, or multi-site external validation. Therefore, the present findings do not support direct transfer, clinical use, or evidence-based implementation in China’s National Fitness Program. Any China-specific use would first require prospective local data collection, culturally and linguistically validated subjective measures, harmonized device and training-zone protocols, local recalibration, athlete- and site-level external validation, and governance safeguards preventing unsupported use for selection, scholarship, employment, or punitive training decisions.

## 6 Conclusion, limitations, and future research

The primary conclusion is that the gated temporal model did not demonstrate above-chance discrimination when temporal adjacency between prediction targets was removed. In the non-overlapping evaluation, DS-MTCN-IG achieved a mean ROC-AUC of 0.469. This finding indicates that the weak discrimination observed in the full-window analysis was not robust and may have been influenced by the extensive overlap between adjacent seven-day histories rather than by a stable athlete-independent training-load signal.

The full-window analysis produced only weak discrimination, near-naïve probability accuracy, very low precision, and no statistically demonstrated predictive advantage of DS-MTCN-IG over logistic regression. The ungated dual-stream model also outperformed the gated architecture across the five neural-network seeds, so the selective-fusion hypothesis was not supported. Integrated-Gradients rankings were moderately consistent at a broad level but unstable for individual top-ranked inputs. The evidence therefore does not support temporal deep learning as a robust or practically useful classifier for this dataset.

The study’s main contribution is a methodological benchmark demonstrating how participant grouping, temporal overlap, calibration, conventional baseline comparison, stochastic variation, and observation-process information can materially change the interpretation of longitudinal sports-injury models. Prospective multicentre validation with clinically verified outcomes and genuinely prospective prediction windows would be required before any applied or decision-support use could be considered.

Several limitations should be explicitly noted. First, the study does not predict overuse injuries, clinical diagnoses, anatomical injury sites, onset mechanisms, or injury severity because these details are absent from the source label. Second, the data come from a single Dutch competitive running team, so external validity to other endurance-sport contexts, including China, is unknown. No external validation was performed. All performance estimates represent internal athlete-disjoint validation within a single Dutch competitive running team and should not be interpreted as evidence of transportability to other teams, sports, performance levels, age groups, sexes, geographic regions, climates, competition calendars, medical-surveillance systems, questionnaire languages, or device protocols. Although complete athletes were held out during cross-validation, all athletes originated from the same organizational and measurement environment. External validity therefore remains unestablished, and local recalibration alone may be insufficient if predictor distributions, recording practices, or predictor–outcome relationships differ across settings. Third, 42,766 rows do not represent 42,766 independent participants; the effective independent sample is limited by the 74 athletes and their unequal follow-up. Fourth, important determinants such as prior injury, sex, age, event specialty, biomechanics, sleep, nutrition, pain, menstrual health, treatment, and environmental exposure were unavailable. Fifth, subjective-rating availability was informative, indicating potential observation-process leakage rather than purely physiological signal. Sixth, the common reporting threshold was used only for descriptive comparison and was not linked to clinical utility, decision costs, or intervention outcomes. Because injury onset and duration were unavailable, the seven-day predictor histories may include post-injury rest or altered reporting behavior for multi-day injury episodes, creating possible reverse causality or outcome-proximity information. The dataset also lacks an active comparator condition or prospective intervention phase; therefore, differences between injury-labeled and non-injury-labeled windows may reflect training participation, reporting behavior, surveillance practices, or unmeasured clinical context rather than a separable physiological load mechanism. Finally, the study was retrospective and did not evaluate prospective impact, fairness, behavioral response, or alert fatigue. The primary full-window evaluation contained extensive overlap between adjacent seven-day histories. Although complete athletes were held out across outer folds, temporal overlap within each athlete reduced the effective information content of the row-level dataset. The collapse of gated-model discrimination in the non-overlapping sensitivity analysis indicates that the primary estimate was sensitive to temporal adjacency and should not be interpreted as stable forecasting performance. The approximately seven-year observation period may contain unmeasured seasonal structure associated with competition schedules, training phases, weather, daylight, surface conditions, holidays, and medical-surveillance practices. Excluding the raw date index from the predictor set does not eliminate seasonal confounding because training-load patterns can encode season indirectly. Calendar dates, competition schedules, and environmental measurements required to separate these effects were unavailable. Integrated-Gradients summaries were based on a deterministic subset of 100 injury-labeled held-out records per seed rather than all 583 injury-labeled observations; consequently, exact attribution rankings should be interpreted as a model-audit summary of the explained subset rather than a cohort-wide feature hierarchy.

Future research should begin with prospectively registered, multicenter cohorts using a consensus injury definition and independent clinical outcome verification. Participating centers should include different teams, performance levels, sexes, age groups, geographic regions, and seasonal training environments. Sample-size planning should be based on the number of independent athletes and injury-labeled observations required for precise discrimination and calibration estimates rather than the nominal number of athlete-day rows. Athlete-level and site-level separation should be preserved through leave-one-site-out or temporal-external validation.

Prospective datasets should additionally record anatomical diagnosis, onset mechanism, severity, time loss, prior injury, pain, event specialty, sex, age, training phase, competition schedule, weather, surface, sleep, nutrition, menstrual-health variables, treatment, and athlete availability. Physiological and biomechanical measurements should be added only after measurement reliability and incremental predictive value have been prespecified. Before applied use, future studies should evaluate calibration transportability, decision-curve net benefit, false-alert burden, fairness, behavioral effects, and whether model-assisted decisions improve athlete outcomes compared with standard monitoring.

Explanations should be checked for stability in folds, seeds and baselines and counterfactual claims should not be used unless interventions can be identified. Add wearable features only by incremental-value analyses pre-specified. Most important, future trials should assess the impact of using the model on decisions and outcomes without adding undue training restrictions.

The overall take-away message is that responsible modeling requires alignment among the endpoint, available predictors, validation design, performance metrics, explanations, and claims. In this dataset, explainable temporal deep learning is most defensible as a methodological audit and feasibility benchmark. It did not establish a robust athlete-independent predictive signal; instead, it showed how temporal overlap, observation-process information, and validation design can alter apparent model performance and transportability claims. These findings do not justify automated injury prevention, clinical monitoring, or intervention deployment; they provide a cautious foundation for better-designed prospective studies.

## Data Availability

The original contributions presented in the study are included in the article/Supplementary material, further inquiries can be directed to the corresponding author.
